# Selection of medicinal plants for traditional medicines in Nepal

**DOI:** 10.1186/s13002-021-00486-5

**Published:** 2021-10-16

**Authors:** Durga H. Kutal, Ripu M. Kunwar, Yadav Uprety, Yagya P. Adhikari, Shandesh Bhattarai, Binaya Adhikari, Laxmi M. Kunwar, Man D. Bhatt, Rainer W. Bussmann

**Affiliations:** 1 University of Wisconsin-Whitewater, Whitewater, WI USA; 2 Ethnobotanical Society of Nepal, Kathmandu, Nepal; 3 Amrit Science College, Tribhuvan University, Kathmandu, Nepal; 4 University of Bayreuth, Universitätsstr. 30, 95447 Bayreuth, Germany; 5 Nepal Academy of Science and Technology, Khumaltar, Nepal; 6 Institute of Forestry, Tribhuvan University, Pokhara, Nepal; 7 Tribhuvan University, Kathmandu, Nepal; 8 Siddhanath Science Campus, Tribhuvan University, Mahendranagar, Nepal; 9 Institute of Botany, Ilia State University, Tbilisi, Georgia

**Keywords:** Medicinal plants, Moraceae, Underutilized, Over-utilized, Binomial regression

## Abstract

**Background:**

There are handful hypothesis-driven ethnobotanical studies in Nepal. In this study, we tested the non-random medicinal plant selection hypothesis using national- and community-level datasets through three different types of regression: linear model with raw data, linear model with log-transformed data and negative binomial model.

**Methods:**

For each of these model, we identified over-utilized families as those with highest positive Studentized residuals and underutilized families with highest negative Studentized residuals. The national-level data were collected from online databases and available literature while the community-level data were collected from Baitadi and Darchula districts.

**Results:**

Both dataset showed larger variance (national dataset mean 6.51 < variance 156.31, community dataset mean 1.16 < variance 2.38). All three types of regression were important to determine the medicinal plant species selection and use differences among the total plant families, although negative binomial regression was most useful. The negative binomial showed a positive nonlinear relationship between total plant family size and number of medicinal species per family for the national dataset (*β*1 = 0.0160 ± 0.0009, *Z*1 = 16.59, *p* < 0.00001, AIC1 = 1181), and with similar slope and stronger performance for the community dataset (*β*2 = 0.1747 ± 0.0199, *Z*2 = 8.76, *p* < 0.00001, AIC2 = 270.78). Moraceae and Euphorbiaceae were found over-utilized while Rosaceae, Cyperaceae and Caryophyllaceae were recorded as underutilized.

**Conclusions:**

As our datasets showed larger variance, negative binomial regression was found the most useful for testing non-random medicinal plant selection hypothesis. The predictions made by non-random selection of medicinal plants hypothesis holds true for community-level studies. The identification of over-utilized families is the first step toward sustainable conservation of plant resources and it provides a baseline for pharmacological research that might be leading to drug discovery.

**Supplementary Information:**

The online version contains supplementary material available at 10.1186/s13002-021-00486-5.

## Introduction

Selection of plants for specific ethnobotanical uses follows two main pathways: (1) random selection, where no regard is taken of the taxonomic affinities, ecological clues, ethnobotanical context or other intrinsic qualities; and (2) targeted or focused selection based on ecological traits (plants in particular habitats with particular growth habits, conservation priorities), or ethnopharmacological appraisals (identifying plants used traditionally to target specific diseases) [1, 2]. It is assumed that the selection of medicinal plants in traditional pharmacopeias is non-random and influenced in part by therapeutic efficacy [3], in part by social and cultural factors [4–6] and in part by taxonomic affiliation [7, 8].

In 1979, Moerman [3] tested the "non-random hypothesis of medicinal plant selection" which predicts that large families are more likely to be richer in medicinal plants than small-sized families. The hypothesis implies that medicinal plants are not randomly selected by local communities, so that a linear positive relationship can be expected between the number of medicinal plants in a family and the size of the family [9]. Because of this non-random selection, some plant families tend to be over- or underrepresented in a given pharmacopeia [9–11]. This implies that "plant family" can become a strong determinant of plant use value [12]. One important question that can be appraised in this connection is why some plants in a particular family are predominantly used or over-utilized in some pharmacopeias and in some regions while other plants are underutilized?

To test the idea that traditional medical systems are influenced in part by therapeutic efficacy, Moerman [3] linearly regressed the number of medicinal plant species per family against the total number of species per family. Despite debated [8, 13], this method has been frequently used and tested in several geographic contexts, e.g., in Amazonian Ecuador [8, 13], in Belize [14], in Kashmir, India [15], in Hawai’i, USA [11], in Pakistan [16], in Mexico [4], in South Africa [9, 17] and in Italy [18]. Nonetheless, such hypothesis-driven ethnobotanical studies are scant particularly in plant-rich countries with broad traditional medicinal knowledge like Nepal [6, 19–23]. Recent studies still focus on medicinal plant diversity, their use patterns and conservation issues [24–26]. In this study, we tested the non-random medicinal plant selection hypothesis through using national- and district-level datasets. These two dataset helps compare the results and factors influence the selection of medicinal plants at national and local level. The latter dataset collected from particular ethnic groups of the northwestern mountainous districts of Nepal served to gauge the influence of sociocultural reasons for medicinal plant selection whereas the former dataset was exclusively random and it served to scrutinize the relationship between the number of medicinal plants in families and the size of those families.

## Materials and methods

### Study sites description

Nepal occupies about 0.1% of earth’s terrestrial land, but it harbors 3.2% of the world’s known flora [27]. So far, 13,067 plant species have been described from Nepal [28], which includes 41 species of gymnosperm [29], and about 7000 species of flowering plants [28, 30], of which 2500 species are used medicinally [31]. The medicinal use of plants in Nepal covers 3000 years of Ayurvedic use and a longer tradition of conservation for subsistence, household economy, primary health care and culture of indigenous people [19, 32–34]. Socioculturally, the country has over 125 ethnic groups with castes including Brahmin, Chhetri, Chepang, Gurung, Magar, Raute, among others [35]. The former two are the dominant ethnic groups in our study sites as well as dominant in the country. There are about 16% Chhetri, 13% Brahmin, 13% Dalit (disadvantaged groups), 36% ethnic groups and 22% other groups and castes in Nepal [36]. The study districts Baitadi and Darchula represent the lower and southern part of the Kailash Sacred Landscape bordering China to the north and India to the west, are dominated by Chhetri about 60% followed by Brahmin 20%, Dalit 10% and others 10% [37]. Our sample population of the two districts represents Chhetri 58%, Brahmin 14%, Dalit 4% and others 24%.

**Fig. 1:**
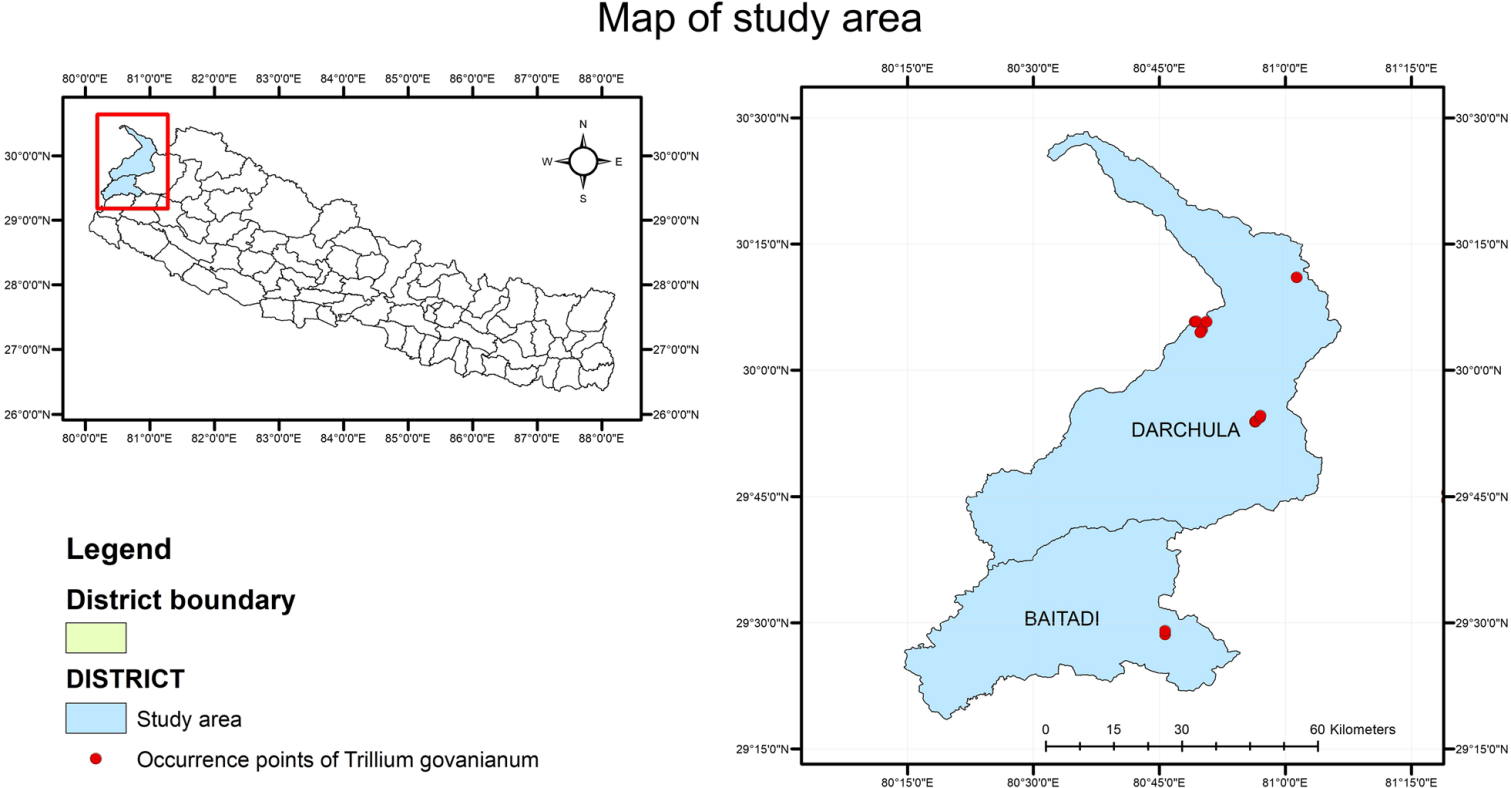
Study area and sites

Much of the area consists of dry, steep, semiarid and alpine rugged terrain [38]. These rangelands intergrade into temperate and subtropical forests, agricultural fields, river valleys and human populated villages [39, 40]. Forest types of the area range from tropical Sal (*Shorea robusta* Gaertn.) forest to alpine *Betula–Rhododendron* [41] and *Juniper–Anthopogon* scrubs. The bioclimate ranges from subtropical in the Baitadi district to alpine in the higher reaches of the mountainous Darchula district [42]. The upper Darchula district is originally known for growing Amaranth [43] and is a part of the relict hemp culture [44]. The area is popularly known for a variety of medicinally important species, which are used for primary health care in the region and also highly valued in other parts of Nepal and in India, Tibet and China [45] (Fig. 1).

### Data collection

For this study, we used two variables: the total number of recorded species per family and the total number of medicinal plant species recorded per family (count data) at national level and community level. The national-level data were collected and adapted from an online database (efloras.org) and other literature [29, 31, 34, 46–49]. For community-level data on the floristic composition and useful medicinal plants of Baitadi and Darchula (BD) districts, intensive three-year fieldwork was conducted by the second author between 2016 and 2018 [37]. A total of 100 participants (57 from Baitadi and 43 from Darchula, 68 men and 32 women) representing traditional healers, plant collectors and traders, and elderly people of ages 40–102 were consulted for interviews following snowball sampling. Conversations with healers and elders were based on a common objective: to increase knowledge regarding herbal remedies and extend educational materials of local interest, as suggested in the guidelines of the International Society of Ethnobiology Code of Ethics [50]. Plant families follow the plant list theplantlist.org. Lowest taxon used for this study was species. Subspecies were not accounted. Research permission was granted by the Institutional Review Board, Florida Atlantic University, USA, and prior informed consent was obtained from the division forest office Baitadi and Darchula districts, Nepal and all interview participants.

### Data analysis

Some earlier studies employed the contingency table [8], least squares regression [9] and Bayesian analysis [51–53] to explore the relationships between the number of known medicinal plants in families (dependent variable) and the size of the family (independent variable). In this study, we used three statistical approaches considering a total of 231 plant families (sample set 1, *n*1 = 231) for the national-level assessment and 105 plant families (sample set 2, *n*2 = 105) for the district or community-level assessment. First, we fitted the simple linear model (LM model 1) to the untransformed data as commonly done in previous studies [3, 7, 14]. In model 2, we fitted the general linear model to the log + 1-transformed model (LogLM) as done in a study [11]. Finally, as LM and LogLM reveal poor performance in modeling count data [54], we fitted generalized linear model with negative binomial (NB model 3) following Robles et al. [13] and Muleba et al. [17].

We fitted a NB model to the medicinal plant data collected while also fitting the simple linear model with both untransformed and log-transformed data for comparison purpose. For each of these models, we identified over-utilized families as those with positive residuals, meaning that these families contained a higher number of recorded medicinal species than would be expected from the model fitted. To identify the most over- and underutilized medicinal plant families, we used the Studentized residuals instead of the raw residuals [18]. Because raw residuals do not have a scale, it is difficult to determine what constitutes large or small residuals. Studentized residuals are often used to find outliers because they follow Student's *t*-distribution with *n-k-2* degrees of freedom, where *n* is the number of observations and *k* is the number of regressors [55, 56]. All analyses were done in R (R Development core Team 2016).

## Results

**Fig. 2:**
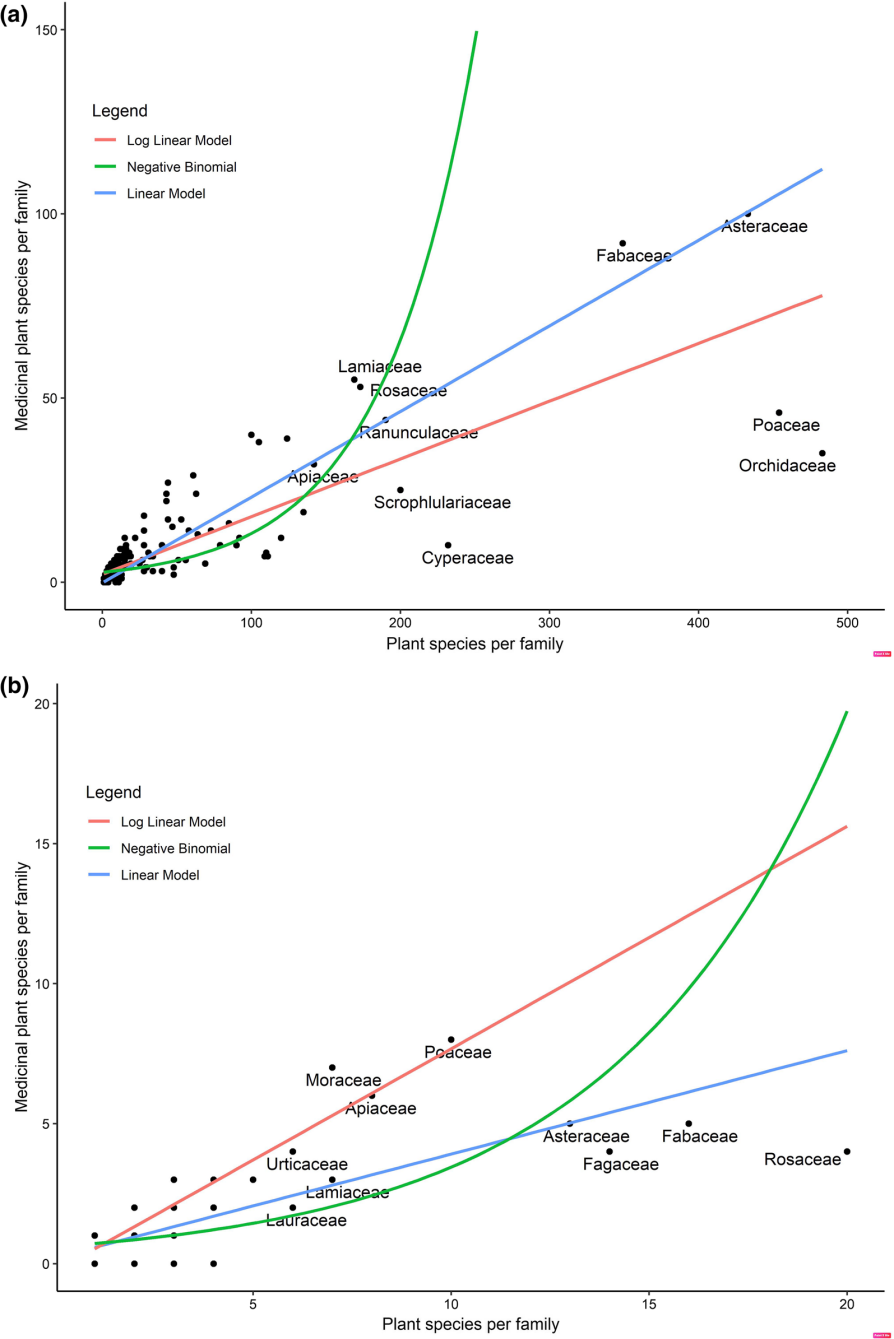
Relationships between number of medicinally used plants and the total number of plants per family in Nepal (**a**) and in Baitadi and Darchula districts (**b**)

From the sample dataset of flowering and medicinal plants of Nepal, we recorded only ~ 28% and ~ 48% plants as medicinal in the national (*n*1)- and district (*n*2)-level datasets. A total of 6526 plant species and 1506 medicinal species was recorded under 231 families from the national-level data, and 255 plant species with 122 medicinal species of 105 families were reported from the district-level local data (Additional file 1). Our analysis revealed that some plant families were over-utilized, while others were underutilized. In the national data, the upper half of the families (115) with descending order of species harbored 1383 medicinal plant species (12 species/family) whereas the lower half (116) contributed only 118 medicinal plant species (1 species/family). The largest 27 families contributed over half of the medicinal plant species (*n* = 753). The top five families namely Orchidaceae, Poaceae, Asteraceae, Fabaceae and Cyperaceae contributed 283 medicinal plant species (56.6 species/family). A total of 45 plant families contained no species used medicinally (Fig. 2).

All three methods were important to determine the medicinal plant species use differences among the total plant families, although the NB regression model was most useful for our datasets as our datasets showed larger variance (national dataset mean 6.51 < variance 156.31, BD dataset mean 1.16 < variance 2.38). The mean number of medicinal plant species per family was 1.016 = exp (0.0160) and 1.190 = exp (0.1747) for the national and local or community or BD datasets respectively. The NB regression model showed a significant positive nonlinear relationship between total plant family size and number of medicinal species per family for the whole Nepal data (*β*1 = 0.0160 ± 0.0009, *Z*1 = 16.59, *p* < 0.00001, AIC1 = 1181), with similar slope for the community-level data (*β*2 = 0.1747 ± 0.0199, *Z*2 = 8.76, *p* < 0.00001, AIC2 = 270.78) (Fig. 2). The results of AIC2 < AIC1 show that community-level data performed stronger in modeling than the national-level data. Accordingly, the Studentized residuals followed a *t*-distribution with 228 degrees of freedom (*n-k-2*) for the national dataset and 102 df for community-level dataset. The 5% critical value of the national dataset was *t*0.05(2), 228d.f. = 1.97 and for the community dataset *t*0.05(2),102d.f. = 1.983. Community data possessed less ranged residuals (+ 4.5 to − 4.66) than that of national data (+ 5.5 to − 7.52) in linear model regression (Additional file 2, 3). The NB model residual values ranged from + 4.24 to − 1.25 for national data and + 3.73 to − 2.56 for community-level data. Families with large positive residuals are over-utilized and, while families with large negative values are used less than chance would allow.

**Fig. 3:**
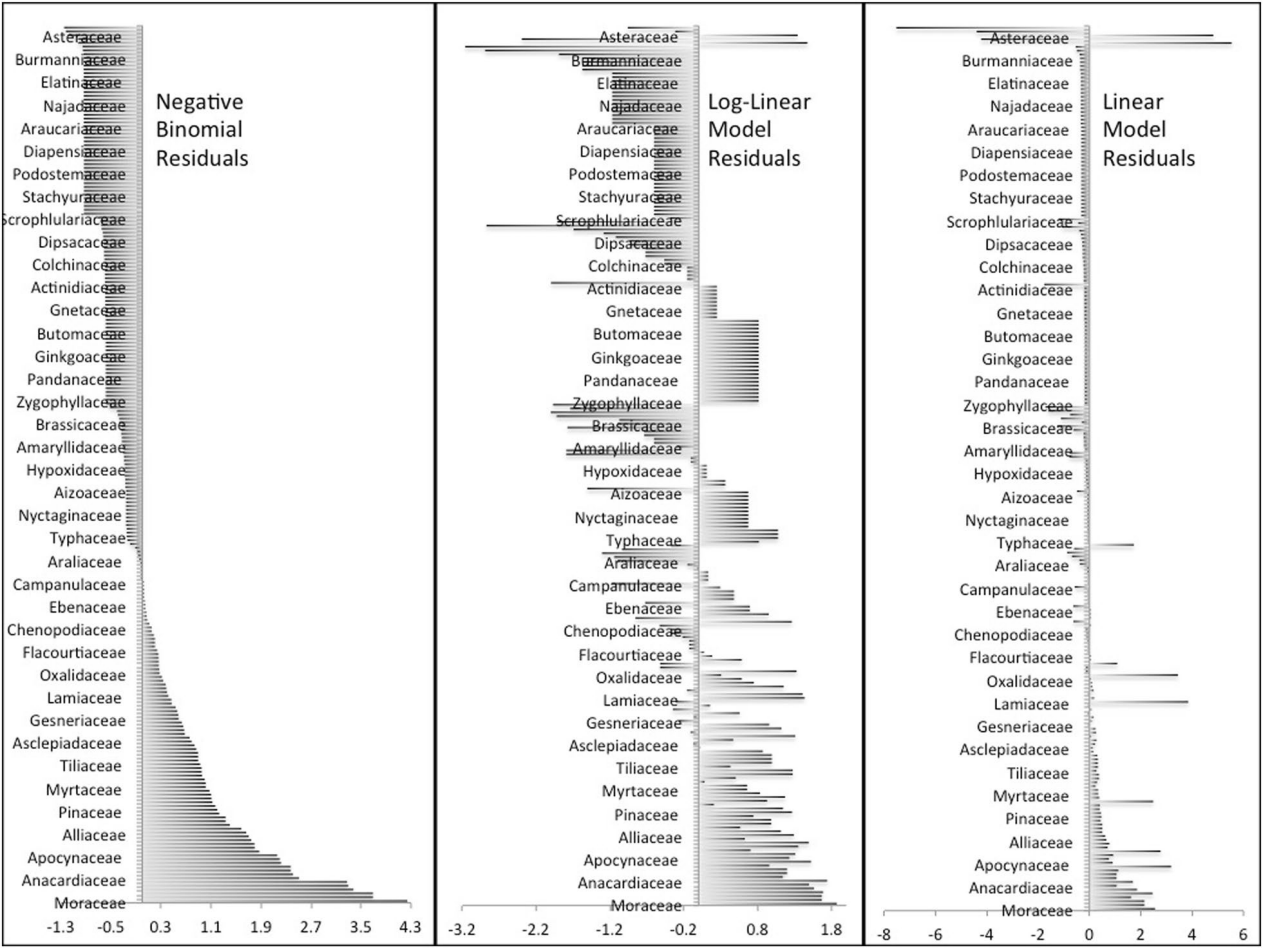

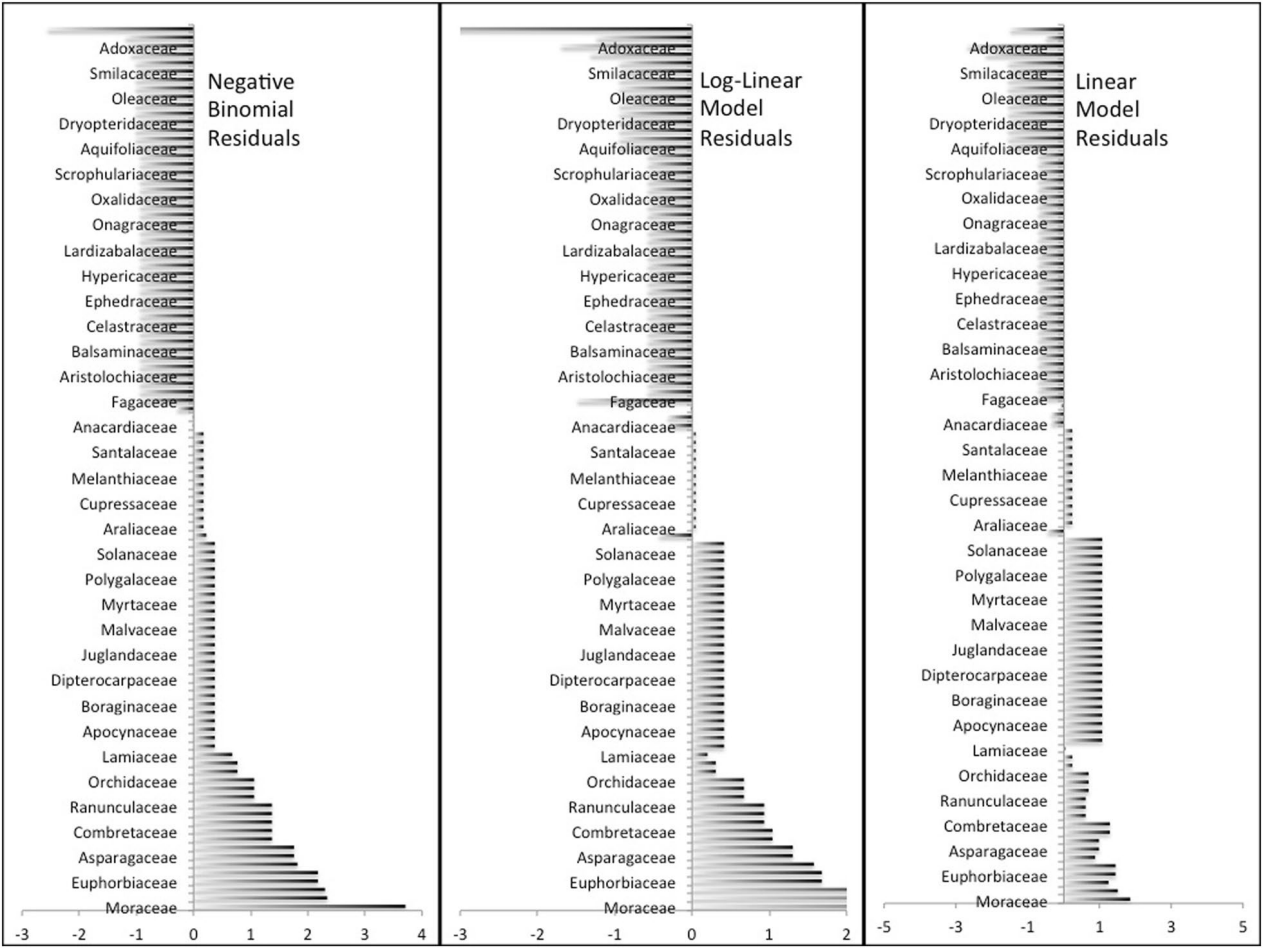
Studentized residuals of all three models applied to two datasets (**a** national and **b** community) showing over-used and underused families

For the whole national dataset, in the NB generalized linear model, 13 plant families had residual values above the 5% critical value: Moraceae (residual = +4.24), Cucurbitaceae (+ 3.69), Zingiberaceae (+ 3.69), Rutaceae (+ 3.37), Solanaceae (+ 3.29), Malvaceae (+ 3.27), Anacardiaceae (+ 2.60), Araceae (+ 2.41), Amaranthaceae (+ 2.37), Oleaceae (+ 2.37), Verbenaceae (+ 2.22), Euphorbiaceae (+ 2.20) and Apocynaceae (+ 2.15). The logLM conforms top seven over-utilized families but in a slightly different sequence: Moraceae (+ 1.87), Anacardiaceae (+ 1.74), Rutaceae (+ 1.68), Zingiberaceae (+ 1.66), Cucurbitaceae (+ 1.66), Solanaceae (+ 1.56), Euphorbiaceae (+ 1.52), Malvaceae (+ 1.49). The top seven over-utilized families in simple LM (Moerman’s approach) appeared in a quite different set (Fabaceae (+ 5.54), Asteraceae (+ 4.82), Lamiaceae (+ 3.85), Rosaceae (+ 3.44), Euphorbiaceae (+ 3.18), Polygonaceae (+ 2.77) and Moraceae (+ 2.55). The over-utilized families (*t* ≥ 1.983) from all three models based on local district-level data showed that the over-used families were generally in similar order with some slight differences. Top over-utilized families in LM, LogLM and NB models were Moraceae (NB + 3.73, LM + 4.61, LogLM + 1.85), Poaceae (+ 2.35, + 4.57, + 1.51), Apiaceae (+ 2.31, + 2.96, + 1.25), Euphorbiaceae (+ 2.18, + 1.68, + 1.44) and Meliaceae (+ 2.18, + 1.68, + 1.44) (Fig. 3).

We found no underutilized families (< −1.97 residual value in NB model) in the national dataset. However, underutilized families in NB model and LM comprise Fabaceae (− 1.02), Cyperaceae (− 1.03), Asteraceae (− 1.18), Poaceae (− 1.22) and Orchidaceae (− 1.25), and Saxifragaceae (− 1.71), Caryophyllaceae (− 1.76), Cyperaceae (− 4.22), Poaceae (− 4.39) and Orchidaceae (− 7.52), respectively. According to Moerman [57], a Studentized residual less than − 1 or greater than 1 indicate significance. In LogLM the underutilized families were Caryophyllaceae (− 1.99), Aquifoliaceae (− 2.31), Cyperaceae (− 2.39), and Juncaceae (− 2.87) and Sabiaceae (− 2.89) with only two common families Cyperaceae and Caryophyllaceae in both models. The NB model for community-level data showed Rosaceae (− 2.56) as the only significantly underutilized family in districts (< − 1.983). Other underutilized families in the NB model were Fabaceae (− 1.19), Adoxaceae (− 1.17), Primulaceae (− 1.09) and Thymelaceae (− 1.01), and the LM confirmed these as the top underutilized families, but in a slightly different sequence: Rosaceae (− 4.66), Fabaceae (− 1.22), Adoxaceae (− 1.69), Primulaceae (− 1.31) and Thymelaceae (− 0.94). The logLM showed the top four underutilized families as Adoxaceae (− 2.67), Primulaceae (− 2.17), Thymelaceae (− 1.55) and Rosaceae (− 1.47) (Fig. 3).

## Discussion

### Catalogue of medicinal plants

The number of flowering and medicinal plants documented in this study (6526) was less than the original estimates [28, 29, 48, 58]. The real proportion of medicinal plants is likely greater than what we report here. This underreporting could be due to the following reasons i) there exists a very limited number of extensive field-dedicated ethnobotanical surveys in Nepal, and ii) the identification of voucher specimens in Nepal is still limited, given that taxonomic experts and resources are limited. Moreover, a comprehensive flora of Nepal is still unavailable [59] which constrains the database and analysis. In order to define if plants are preferentially selected or avoided it is necessary to have a complete and up to date flora of the area [18]. The use of plant databases and the associated knowledge of plant uses to formulate and test theories and hypotheses in ethnobotany is not yet a common practice despite the recent calls for more hypothesis-driven ethnobotanical researches [17]. The paradigm shift toward a more hypothesis- or theory-driven ethnobotany is important to make ethnobotany a stronger scientific discipline with theories and hypotheses that can be used to predict new medicinal plant uses as well as better explain plant–human interactions [60, 61].

### Underutilized medicinal plants

Various plant families with numerous species, were found as not selected for medicine, while other less abundant families contained many medicinal species. The large families such as Orchidaceae, Asteraceae, Fabaceae, Poaceae and Cyperaceae were found to be used less frequently in Nepal. The underutilization of Poaceae, Orchidaceae, Cyperaceae and Fabaceae is consistent with the earlier reports [13, 15, 16, 51–53, 57, 62]. Our findings of significant underused (Poaceae, Orchidaceae, Cyperaceae and Fabaceae) and over-used (Moraceae, Solanaceae, Cucurbitaceae and Malvaceae) plant families significantly overlapped with a study from Campania, Italy [51]. This could be attributed to the fact that both areas are characterized by hill and mountainous physiography. Highly preferred fodders in hilly areas of Nepal came from Moraceae [63].

Certain plant families contain chemical compounds (often serving as chemical defense) that are more useful or effective as medicines, while other families are much less useful as medicines (e.g., Poaceae, Cyperaceae, given that they often depend on resprouting and physical defenses rather than chemical defenses). Because of these characteristics, Cyperaceae and Poaceae are underutilized [11]. A high percentage of flavonoids in Anacardiaceae and terpenoids in Euphorbiaceae [9] might correlate with their over-utilization in the Nepalese pharmacopeia. Fabaceae was over-utilized in LM and logLM models, while the NB model showed it as underutilized, consistent with the findings of Muleba et al. [17], indicating a potential over-estimation of medicinal values of some taxa of Fabaceae. This implies that other families may outcompete Fabaceae in terms of people’s preferences for medicinal uses. The Fabaceae is a large, economically and medicinally important family of flowering plants [64], with many documented uses, and is underutilized in North America and over-utilized for medicine in Korea and Ecuador [57].

### Over-utilized medicinal plants

The recent publications of Robles et al. [13] and Muleba et al. [17] also employed the NB model that we used in our study. Our study showed similarities to theirs, given that in all studies the relationships between medicinal plants and the total flora were not linear as suggested in Moerman [10]. At a 5% level of significance, in both NB and logLM models, we found Moraceae, Cucurbitaceae, Zingiberaceae, Rutaceae, Solanaceae, Malvaceae, Anacardiaceae, Amaranthaceae and Euphorbiaceae as top over-used families. Of these, Moraceae, Zingiberaceae, Cucurbitaceae, Solanaceae, Euphorbiaceae, Malvaceae, and Amaranthaceae have previously been reported as over-utilized [8, 11, 13, 15, 51]. The most over-used family was Moraceae, consistent with the findings of Weckerle et al. [51]. Rutaceae and Anacardiaceae were novel reports as over-utilized families, underlining the fact that these families have therapeutic value, given that they have independently been discovered and adapted in unrelated pharmacopeias [65]. Malvaceae and Euphorbiaceae were listed as being medicinally most important families in the world [66]. As suggested by Moerman et al. [57], ethnographic data are important for the interpretation of trends through patterns observed in exploring the non-random plant selection hypothesis. A non-random selection pattern also provides evidence for the validity of folk therapies and potential efficacy [53]. This asserts that there is a need to apply the most appropriate model while testing ethnobotanical hypotheses. This is paramount because the identification of over- and underutilized families is a first step toward sustainable use, conservation of plant resources and pharmacological studies that might advance pharmacology [65].

### Culture, environment and use pattern

We found a pattern that some medicinal families were over-utilized, i.e., they contained more medicinal plants than expected, whereas others were underutilized, i.e., they had a significantly lower number of medicinal plants. Some large plant families were not selected for medicinal uses, while other less abundant families contained many useful medicinal species. This does not imply that underutilized plant families are not important in ethnomedicine; it rather may be an expression of people’s preferences for medicinal uses. The underutilization of Asteraceae in our study and in Pakistan [16] is a rather interesting result, given the extensive use of Asteraceae and Lamiaceae as medicinal plants reported in other ethnobotanical studies [10, 15, 53]. This result is consistent with the predictions from the non-random selection of medicinal plants hypothesis. Interestingly, the most abundant families are underrepresented in the Nepalese ethnopharmacopeias, supporting the hypothesis that people utilize plants based on traditional knowledge and culture, not random. *Mentha arvensis* L. and *M. piperita* L. (both from Lamiaceae) have common active phyto-constituents: menthol, menthone, α-pinene, iso-menthone and therapeutic properties: stomachic, digestive and colic [67], but they are differently selected. *M. arvensis* was over-utilized and found collected from 17 districts [68–78] for traditional medicine whereas *M. piperita* was reported as ethnomedicinal in only four districts [76, 79–82]. Despite the morphological, and phytochemical resemblance, these two species were selectively collected conforming that the collection is not random, influenced by traditional knowledge.

The over-utilized families did, however, include highly preferred medicinal plant species. Moraceae was over-used medicinally by Nepalese communities. It is also possible that plants in these over-utilized families (Moraceae, Euphorbiaceae, etc.) were also preferred to cultural reasons. As example, at local district level, out of seven species from Moraceae utilized in the districts, three (*Ficus benghalensis* L., *F. palmata* Forssk. and *F. religiosa* L.) were also used for ritual purposes. Moraceae are abundantly grown in anthropogenic landscapes allowing them to be accessed more easily and more frequently, without having to travel long distance. The fig family was recognized as the most useful family for indigenous people in Nepal [83]. Before motorized transportation (and even now in the rural areas), fig trees were planted commonly in public resting places (Chautaras) in order to provide shade. Chautaras were constructed over the course of centuries as four-cornored resting place for travelers especially porters in the hills [84].

The AIC result showed that community-level data revealed a stronger fit to the model than the national-level data. The community-level data have less variance and it could be the reason of homogenous plant collectors and healers (58% Chhetri and 14% Brahmin) in comparison with national-level data. The large positive Studentized residuals values in NB model in national data showed that the plant families are over-utilized; this could be the reason of greater availability of medicinal plants and cultural diversity.

Plants often have uses tied to traditions, religion and ancient cultural practices [18]. Local communities believe that plants become more medicinal when processed spiritually and materially [85]. Community beliefs, rituals and culture are therefore important while utilizing plant resources in sacred landscapes [86]. For example, *Paris polyphylla* Sm. (locally called Satuwa), one of the popular medicinal plants in the region, is used for the treatment of seven ailments (headache, fever, diarrhea, indigestion, wounds, gastritis and snake bites), because people believe that each leaf cures one ailment. Another reason of over-utilization could be due to the availability and abundance of plants in the area. People may be over-utilizing plants that occur in abundance [21, 87]. These findings may support the hypotheses of availability and non-random plant selection.

## Conclusions

Linear model, log-transformed linear model, negative binomial, Bayesian analysis and least square regressions are common methods to test the idea whether the plants are preferentially selected for traditional medicine. The former three were important to determine the use differences in medicinal plant species among all plant families encountered, although negative binomial regression was found most useful, given that our datasets showed larger variance. The analysis showed that large families tend to have more species being considered for local medicinal applications, a salient confirmation of the non-random plant selection for medicinal purposes. However, the different models depicted a different sequence of the families. This study provides evidence that the predictions made by the non-random selection of medicinal plants hypothesis holds true for community-level studies, because most of the over- and underutilized medicinal plant families we identified concurred with results from other studies. Of two datasets, community-level data revealed a stronger fit to the model than the national-level data. This study allows identifying the plant families most important for conservation, pharmacological advancement and promotion of traditional medicines.

## Supplementary Information

**Additional file 1.** Total 255 plant species with 122 medicinal species of 105 families in Baitadi and Darchula districts.

**Additional file 2.** Regression analysis and residuals of 231 families of national level data.

**Additional file 3.** Regression analysis and residuals of 105 families of local district level data.

## Data Availability

All relevant data are within the manuscript and its Supporting Information files.
