# Correction to: Selection of medicinal plants for traditional medicines in Nepal

**DOI:** 10.1186/s13002-021-00491-8

**Published:** 2021-11-08

**Authors:** Durga H. Kutal, Ripu M. Kunwar, Yadav Uprety, Yagya P. Adhikari, Shandesh Bhattarai, Binaya Adhikari, Laxmi M. Kunwar, Man D. Bhatt, Rainer W. Bussmann

**Affiliations:** 1 University of Wisconsin-Whitewater, Whitewater, WI USA; 2 Ethnobotanical Society of Nepal, Kathmandu, Nepal; 3 Amrit Science College, Tribhuvan University, Kathmandu, Nepal; 4 University of Bayreuth, Universitätsstr. 30, 95447 Bayreuth, Germany; 5 Nepal Academy of Science and Technology, Khumaltar, Nepal; 6 Institute of Forestry, Tribhuvan University, Pokhara, Nepal; 7 Tribhuvan University, Kathmandu, Nepal; 8 Siddhanath Science Campus, Tribhuvan University, Mahendranagar, Nepal; 9 Institute of Botany, Ilia State University, Tbilisi, Georgia

## Correction to: J Ethnobiology Ethnomedicine 17:59 (2021) 10.1186/s13002-021-00486-5

Following publication of the original article [1], the authors advised that an incorrect version of Fig. 1 had been provided.

The published article has now been updated with the correct version of the figure and the corrected figure may be found below.

The authors thank you for reading this correction apologize for any inconvenience caused.

**Fig. 1:**
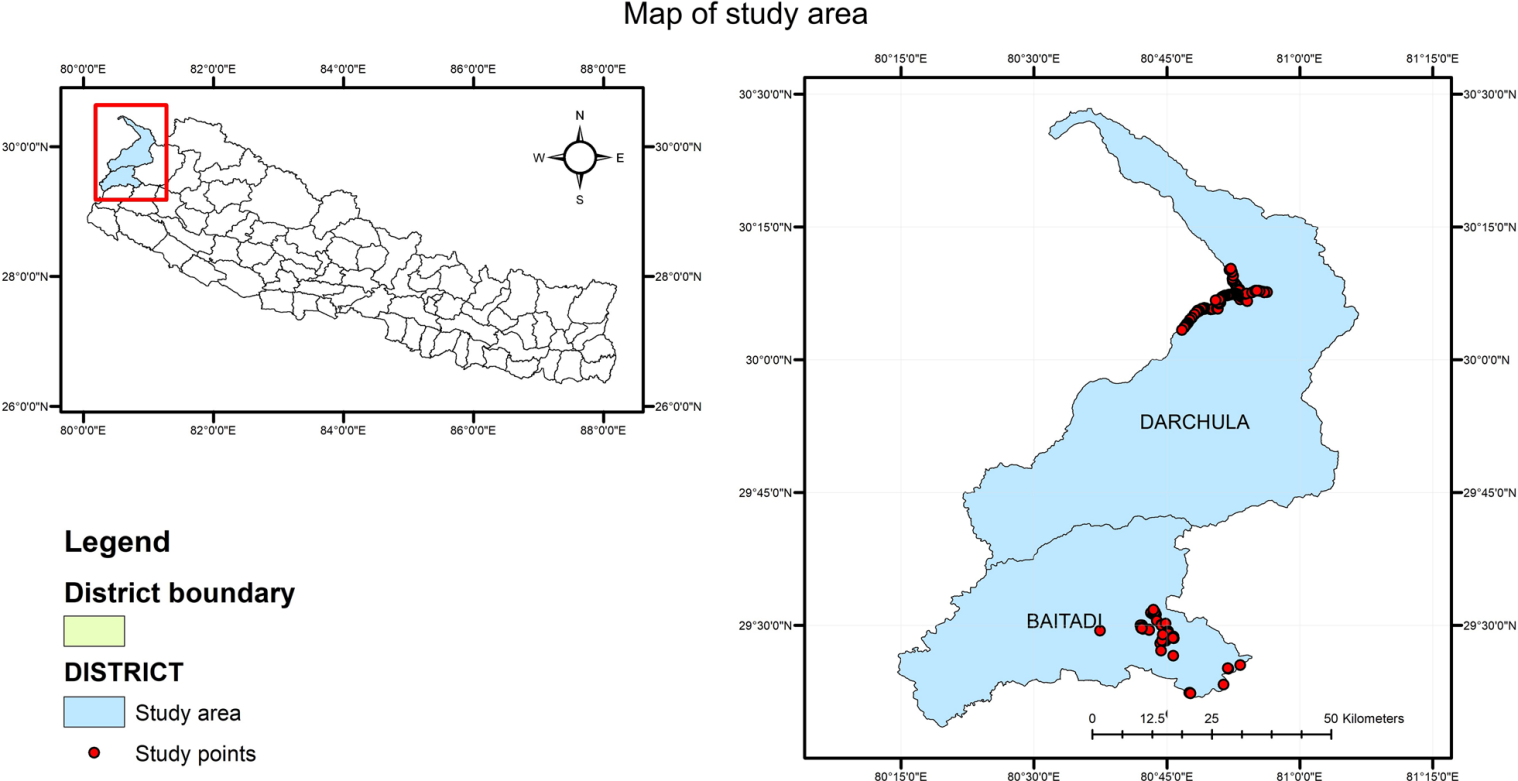
Study area and sites
